# Large-Scale Analyses of Site-Specific Evolutionary Rates across Eukaryote Proteomes Reveal Confounding Interactions between Intrinsic Disorder, Secondary Structure, and Functional Domains

**DOI:** 10.3390/genes9110553

**Published:** 2018-11-14

**Authors:** Joseph B. Ahrens, Jordon Rahaman, Jessica Siltberg-Liberles

**Affiliations:** Department of Biological Sciences, Florida International University, Miami, FL 33199, USA; jba462462@gmail.com (J.B.A.); jraha001@fiu.edu (J.R.)

**Keywords:** evolutionary rates, protein sequence, intrinsic disorder, structural prediction, Eukaryotes

## Abstract

Various structural and functional constraints govern the evolution of protein sequences. As a result, the relative rates of amino acid replacement among sites within a protein can vary significantly. Previous large-scale work on Metazoan (Animal) protein sequence alignments indicated that amino acid replacement rates are partially driven by a complex interaction among three factors: intrinsic disorder propensity; secondary structure; and functional domain involvement. Here, we use sequence-based predictors to evaluate the effects of these factors on site-specific sequence evolutionary rates within four eukaryotic lineages: Metazoans; Plants; Saccharomycete Fungi; and Alveolate Protists. Our results show broad, consistent trends across all four Eukaryote groups. In all four lineages, there is a significant increase in amino acid replacement rates when comparing: (i) disordered vs. ordered sites; (ii) random coil sites vs. sites in secondary structures; and (iii) inter-domain linker sites vs. sites in functional domains. Additionally, within Metazoans, Plants, and Saccharomycetes, there is a strong confounding interaction between intrinsic disorder and secondary structure—alignment sites exhibiting both high disorder propensity and involvement in secondary structures have very low average rates of sequence evolution. Analysis of gene ontology (GO) terms revealed that in all four lineages, a high fraction of sequences containing these conserved, disordered-structured sites are involved in nucleic acid binding. We also observe notable differences in the statistical trends of Alveolates, where intrinsically disordered sites are more variable than in other Eukaryotes and the statistical interactions between disorder and other factors are less pronounced.

## 1. Introduction

Nucleotide substitutions within protein-coding genes can produce downstream changes (amino acid replacements) within the sequences of their translated expression products (proteins). Consequently, protein molecular evolution entails the replacement of amino acid residues at various positions (sites) within a protein’s primary structure (sequence) over time. The relative rates of amino acid replacement may vary significantly among sequence sites, and accounting for rate heterogeneity greatly increases the accuracy of phylogenetic reconstruction based on molecular evolutionary models [1]. This phenomenon has attracted considerable research examining the relationship between protein structure/function and site-specific rates of protein sequence evolution (see Echave et al. [2] for a review).

Several structural and functional properties of proteins are now known to drive overall rates of protein sequence evolution as well as site-specific evolutionary rates within a protein sequence. In particular, sites with a large number of stabilizing contacts (high local packing density) tend to evolve slowly [3,4], and sites with high solvent exposure tend to evolve faster than buried sites [3,5,6]. At the whole-sequence level, there is a strong negative correlation between gene expression level and the rate of protein sequence evolution [7]. Brown et al. [8] also found that proteins with long intrinsically disordered regions (IDRs) tend to experience higher overall levels of amino acid replacement than ordered proteins.

Previously, Ahrens et al. [9] used sequence-based predictors to show that site-specific evolutionary rates in Metazoan (Animal) proteins are partially governed by an interaction among three factors: intrinsic disorder propensity; secondary structure; and functional domain involvement. A strong statistical interaction was detected between conserved intrinsic disorder and conserved secondary structure, and sites which were predicted to be both intrinsically disordered and involved in secondary structures (“disordered-structured” sites) had lower mean rate scores than any other structural category [9,10].

Here, we present an evaluation of the structural factors studied by Ahrens et al. [9] across large-scale protein sequence datasets representing four eukaryotic lineages: Metazoans; Plants; Saccharomycete Fungi; and Alveolate Protists. We used the sequence-based predictors employed in Ahrens et al. [9] on hundreds of thousands of sequences to identify protein family alignment sites with conserved intrinsic disorder, secondary structure and functional domain predictions, and we applied multifactor statistical analyses to measure the effects of these structural/functional factors on site-specific rates of sequence evolution. Despite the moderate error inherent in structural prediction, our results indicate that there are statistically significant, and broadly consistent forces driving eukaryotic protein evolution. Furthermore, proteins with conserved disordered-structured sequence sites are found in all four Eukaryote lineages and appear to be important for nucleic acid binding, as well as various other fold-upon-binding events.

## 2. Materials and Methods

### 2.1. Data Collection

We collected protein sequence data from canonical reference proteomes made available by the UniProt Consortium [11]. These proteomes are useful for evolutionary analysis because, for alternatively spliced genes, only a single protein isoform is chosen to represent each gene locus. We used this data to construct four large-scale protein datasets containing important model organisms from four divergent eukaryotic lineages: Metazoans (Animals), Plants, Alveolate Protists, and Saccharomycete Fungi (see Supplementary Figure S1). To represent Metazoan proteins, we used the 24 Metazoan proteomes (plus the *Monosiga brevicollis* proteome) described in Ahrens et al. [9]. We collected 22 Plant proteomes from the February 2015 release of the UniProt Reference Proteome set, and downloaded two additional proteomes (*Oryza sativa* and *Volox carteri*) directly from UniProt in April of 2016. All of the 44 Alveolate Protist proteomes, as well as the 49 proteomes from Saccharomycete Fungi, were taken from the UniProt Reference Proteome set released in July of 2016. In all four datasets, we excluded any protein sequences that (i) were less than 30 amino acids in length or (ii) contained X characters (indicating missing sequence data) prior to sequence clustering.

### 2.2. Clustering and Multiple Sequence Alignment

Sequence clustering was accomplished by running the graph-based single-linkage program BLASTClust from BLAST v2.2.26 [12] on each of the four datasets described above. We used two criteria (pairwise sequence identity and sequence overlap) to establish linkage: two sequences were grouped in the same cluster if (i) their pairwise sequence identity was at least 40% and (ii) the length of their BLAST alignment footprint (the region of sequence overlap) was at least 90% the length of the longer sequence. The motivation for this permissive clustering approach was to obtain inclusive clusters of homologous protein sequences that were suitable for multiple sequence alignment and subsequent downstream analyses. Clusters containing between 10 and 300 sequences were aligned with MAFFT v7.123b (Animals) and v7.313 (Plants, Protists, Fungi) using the local pairwise alignment strategy and a maximum of 1000 iterations [13]. Sequence alignments were used for downstream evolutionary analysis if the following conditions were met: (i) the minimum pairwise sequence identity (1 − *p*-distance) of any two sequences in the alignment was at least 30%; (ii) every sequence was at least 50% the length of the full sequence alignment; (iii) none of the sequences contained ambiguous characters or non-standard amino acids; (iv) less than 90% of alignment sites were conserved (invariant) at the amino acid level; and (v) at least four sequences in each alignment were unique.

### 2.3. Evolutionary Analysis

We inferred phylogenetic trees using the MPI-enabled version of MrBayes 3.2.2 [14] with tree-bisection-reconnection (TBR) moves disabled. Each analysis used the mixed-model approach (substitution matrix treated as a free parameter) and a four-category gamma distribution among site rates. Analyses were run for 5,000,000 generations, or until the average standard deviation of split frequencies fell below 0.005. Majority-rule consensus trees were constructed for each alignment, discarding the initial 25% of trees as burn-in. To infer site-specific rates of sequence evolution, we used a modified version of the program Rate4site [15] which prints the entire alignment-wide distribution of rate scores rather than only the values associated with a particular reference sequence. Multiple sequence alignments and their associated consensus trees were used as inputs and evaluated under a sixteen-category gamma-distributed model. To more directly measure the values of interest (i.e., the relative site-wise rates of amino acid residue replacement), and in consideration of recent developments in the field [16,17], site rates were scored based on the equal-probability matrix proposed by Jukes and Cantor [18] rather than the default matrix proposed by Jones et al. [19]. We used the empirical Bayesian method of rate inference implemented in Rate4site, and site rates were normalized as z-scores with mean = 0.0 so that in all alignments, positive scores indicated faster sites while negative scores indicated slower sites.

### 2.4. Structural Prediction

As in Ahrens et al. [9], we predicted the intrinsic disorder propensity, secondary structure and functional domains of all sequences in each alignment using sequence-based computational tools. Intrinsic disorder propensity was evaluated using the long disorder prediction method implemented in IUPred 1.0 [20]. The accuracy of IUPred-long varies from 62% against DisProt [21] to 85% against IDEAL [22] using the intended cut-off of 0.5 [23]. However, IUPred has greater accuracy against DisProt using a cut-off of 0.4 [24,25]. Here, sequence sites with a propensity score above 0.4 were considered intrinsically disordered, in accordance with previous studies [9,24,25]. Secondary structures (α-helices, β-strands and random coils) were predicted using PSIPRED 3.4 [26] based on sequence profiles generated with PSIBLAST [27] against a filtered version of the UniRef90 database [28]. Previous benchmarks indicate that when based on sequence profiles, PSIPRED predicts secondary structure with >80% accuracy [29,30]. Functional domains were predicted using the Pfam database [31], and all sequence regions outside of functional domains were considered inter-domain linkers. All binary predictions were mapped onto their corresponding protein family alignment sites, and only alignment sites with conserved predictions were considered for statistical analysis.

### 2.5. Gene Ontology

From each Eukaryote dataset, sequence clusters containing disordered-structured alignment sites (i.e., sites where every sequence in the alignment was predicted to be intrinsically disordered as well as involved in either an α-helix or β-strand) were reserved for gene ontology analysis. Sequences from these alignments corresponding to *Homo sapiens* (Metazoans), *Arabidopsis thaliana* (Plants), *Saccharomyces cerevisiae* (Saccharomycetes) or *Plasmodium falciparum* (Alveolates) were collected and analyzed using the Panther webserver [32,33].

### 2.6. Statistical Analysis

Each alignment site was labelled based on the predicted structural properties of all sequences in the alignment. A site was called “disordered” if the IUPred score for every sequence at that site was above 0.4, and “ordered” if every score was below 0.4. Similarly, a site was considered “structured” if PSIPRED indicated that either (i) every sequence fell within an alpha helix or (ii) every sequence fell within a beta strand, and it was labelled “coil” if all sequences fell within random coils at that site. Finally, sites were called “domain” sites when all sequences fell within a predicted Pfam domain and “linker” sites when none of them fell within a Pfam domain. Sites containing any number of gap characters were excluded from further evaluation.

All statistical analysis and visualization was performed in the R programming language [34,35] as well as the “matplotlib” module [36] available in the Python programming language [37]. In each of the four eukaryotic datasets, nonparametric Mann-Whitney tests were used to compare normalized rates of sequence evolution observed in ordered vs. disordered sites, structured vs. coil sites, and domain vs. linker sites found across all sequence alignments. Additionally, based on the above criteria, many alignment sites could be labeled according to all three structural properties (e.g., disordered/coil/linker). Following a Kruskal–Wallis test, nonparametric multiple pairwise significance tests (α = 0.05) were performed to compare the rate distributions of all factor-level combinations (e.g., disordered/coil/linker vs. disordered/coil/domain) in all four datasets via the “kruskalmc” method available in the “pgirmess” package [38] in R. Using the “car” package developed by Fox and Weisberg [39], these sites were also incorporated into an unbalanced (type III) factorial analysis of variance (ANOVA) with zero-sum contrasts to evaluate the statistical interaction among intrinsic disorder, secondary structure and functional domain involvement. The relationship between cluster disorder content (fraction of disordered alignment sites) and mean rate scores within disordered-structured alignment sites was analyzed via Loess regression and visualized in the “ggplot2” library [40].

## 3. Results

### 3.1. Clustering and Phylogenetics

**Figure 1:**
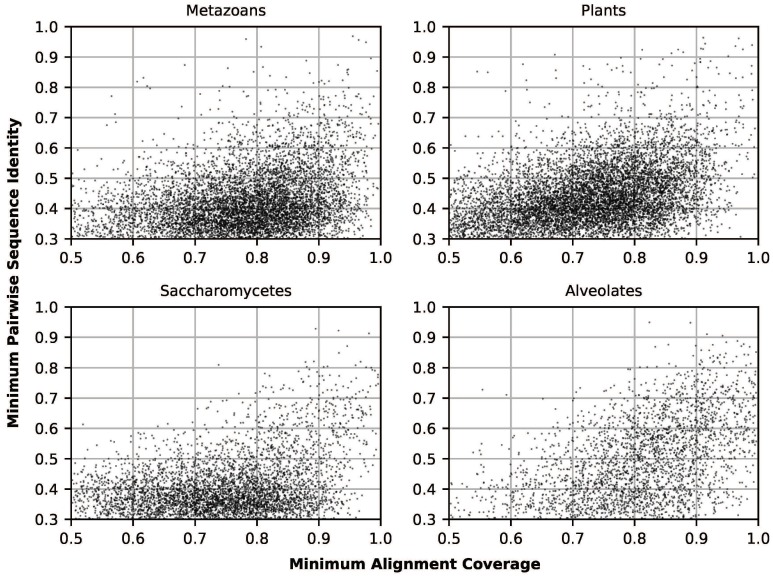
Scatterplots showing minimum pairwise sequence identity (fraction of matching aligned characters) and minimum alignment coverage (seq. length/alignment length) for all Metazoan, Plant, Saccharomycete, and Alveolate clusters used in analyses.

Across all four Eukaryote datasets, single-linkage clustering via BlastClust [12] produced 25,871 clusters containing between 10 and 300 sequences (see Supplementary Figure S1). After multiple sequence alignment, 22,395 (87%) of these clusters were suitable for downstream phylogenetic inference and site-wise evolutionary rate inference (Figure 1; see Methods: Clustering and Multiple Sequence Alignment for suitability criteria). These sequence alignments contained a total of 14,011,483 sites, of which 9,202,935 (66%) contained no gap characters. Refer to Table 1 for more information relating to individual datasets. 

Nearly all of the 22,395 phylogenetic analyses in MrBayes [14] converged in less than 5,000,000 generations. Only 204 (<1%) of the analyses ran for 5,000,000 generations without reaching an average standard deviation of split frequencies (ASDSF) of less than 0.01, the convergence diagnostic value recommended by the program authors [41], while 21,952 (98%) reached an ASDSF of less than 0.005.

### 3.2. Structural Prediction

IUPred results [20] indicated that 847,431 of the 9,202,935 gap-free sites were conserved disordered alignment sites (i.e., sites where every sequence in an alignment was intrinsically disordered) and 5,551,255 were conserved ordered sites. Relative to the number of gap-free sites, the percentages of conserved disordered alignment sites in Metazoans (11.6%), Plants (8.2%), Saccharomycetes (6.4%), and Alveolates (9.7%) were consistently low (see Table 1). PSIPRED [26] indicated 3,216,527 conserved structured sites (sites where every sequence fell within either an α-helix or a β-strand) and 3,474,440 conserved coil sites, and Pfam [31] indicated 3,972,117 conserved domain sites and 4,132,983 conserved linker sites. Furthermore, 4,206,014 sites could be consistently labeled according to all three binary factors (e.g., all sequences predicted to be disordered/coil/linker at a particular site), making them suitable for multiple pairwise comparison and factorial ANOVA.

### 3.3. Statistical Analysis

**Figure 2:**
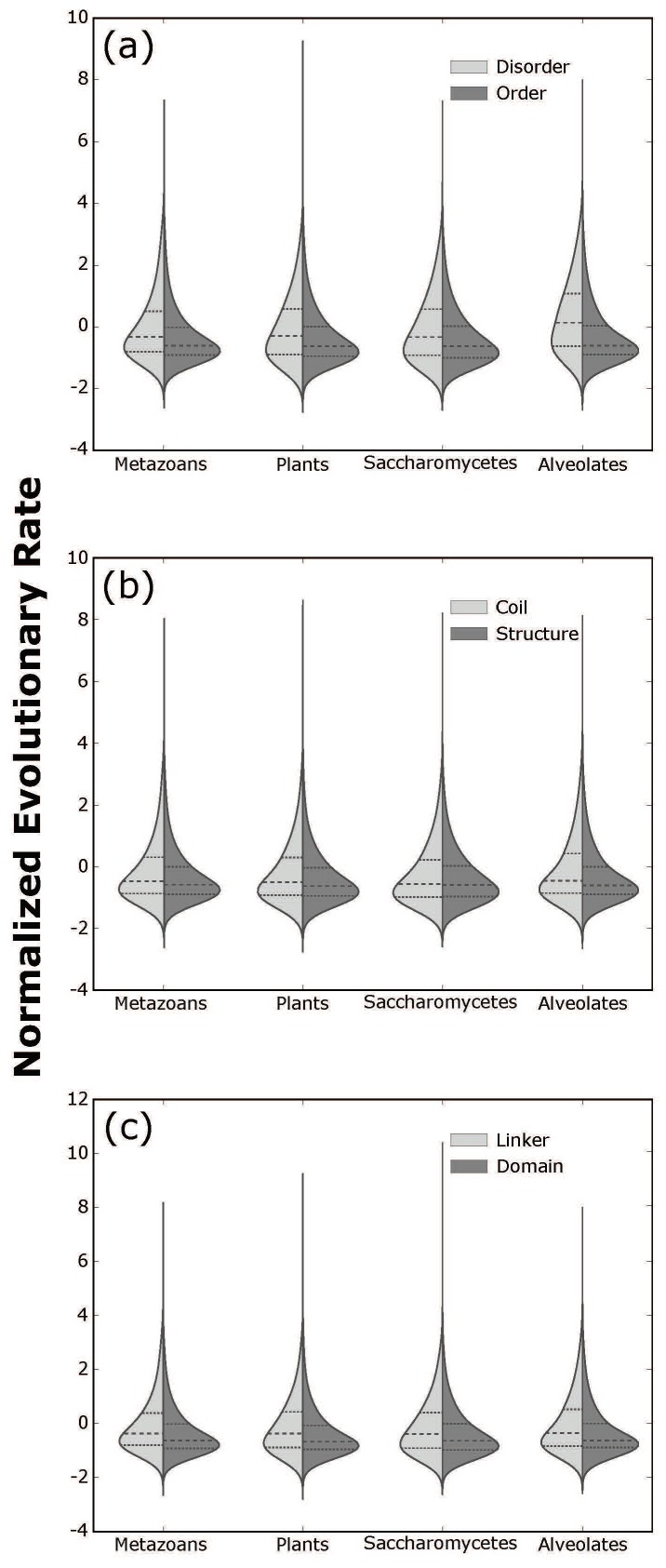
Split violin plots showing differences in normalized site-specific rates of amino acid replacement in: (**a**) ordered vs. disordered sites; (**b**) structured vs. coil sites; and (**c**) domain vs. linker sites within four eukaryotic datasets. Middle dashed lines indicate medians and outer dashed lines indicate quartiles.

Mann-Whitney tests indicated that in all four eukaryotic datasets, disordered sites had higher median amino acid replacement rate scores than ordered sites (Δ_median_rate_ Metazoans: =+0.28, Plants: +0.33, Saccharomycetes: +0.29, Alveolates: +0.75). Similarly, coil sites had higher median rate scores than structured sites (Δ_median_rate_ Metazoans: +0.11, Plants: +0.12, Saccharomycetes: +0.03, Alveolates: +0.15) and linker sites had higher median scores than domain sites (Δ_median_rate_ Metazoans: +0.25, Plants: +0.30, Saccharomycetes: +0.25, Alveolates: +0.27). All median differences in all datasets were highly statistically significant (*p* < 2.2 × 10^−16^), but opposing rate distributions (e.g., order vs. disorder) exhibited large overlaps in their range of values (Figure 2). Notably, Mann-Whitney tests considering only sites from clusters where opposing structural properties co-occur (e.g., disordered and ordered sites found within the same alignment) were statistically significant as well (*p* < 2.2 × 10^−16^). Kruskal-Wallis tests comparing the eight factor-level combinations were statistically significant in all four datasets (*p* < 2.2 × 10^−6^), and most of the 28 post hoc multiple pairwise comparisons were also significant (corrected *p* < 0.05; see Supplementary Table S2).

**Figure 3:**
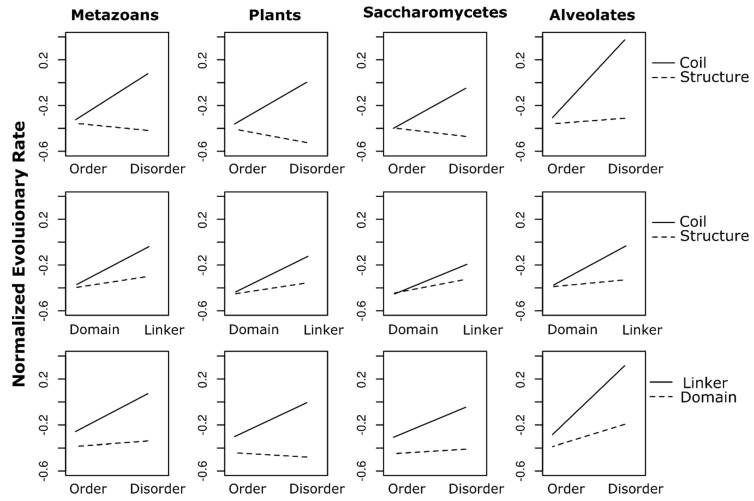
Trace plots illustrating first-order interactions among all site-wise binary factor levels: order (Order) and intrinsic disorder (Disorder), secondary structures (Structure) and random coils (Coil), functional domains (Domain) and interdomain linkers (Linker). Trace factors (solid vs. dashed lines) are indicated to the right of each row of plots. Vertical columns of plots correspond to each of the four datasets (indicated) above. Y-axes represent mean normalized evolutionary rates.

In addition to statistically significant main effects (all *p* < 10^−5^), parametric factorial analyses for all four datasets showed statistically significant interaction terms (all *p* < 2 × 10^−16^). First-order interactions were particularly large between disorder and secondary structure where the effect of disorder was reversed across three of the four datasets: in Metazoans, Plants, and Saccharomycetes, alignment sites predicted to be both disordered and involved in secondary structures (disordered-structured sites) have lower mean rate scores than ordered, structured sites (Figure 3). A similar phenomenon is observed in the disorder x domain interaction in Plants: disordered sites in functional domains tend to be more conserved than ordered domain sites (Figure 3). Higher-order interactions (disorder × structure × domain) were also detected in all four datasets (all *p* < 2 × 10^−16^). Correlation coefficients (adjusted R^2^ values) were low in all four models (Metazoans: 0.04, Plants: 0.03, Saccharomycetes: 0.02, Alveolates: 0.06).

**Figure 4:**
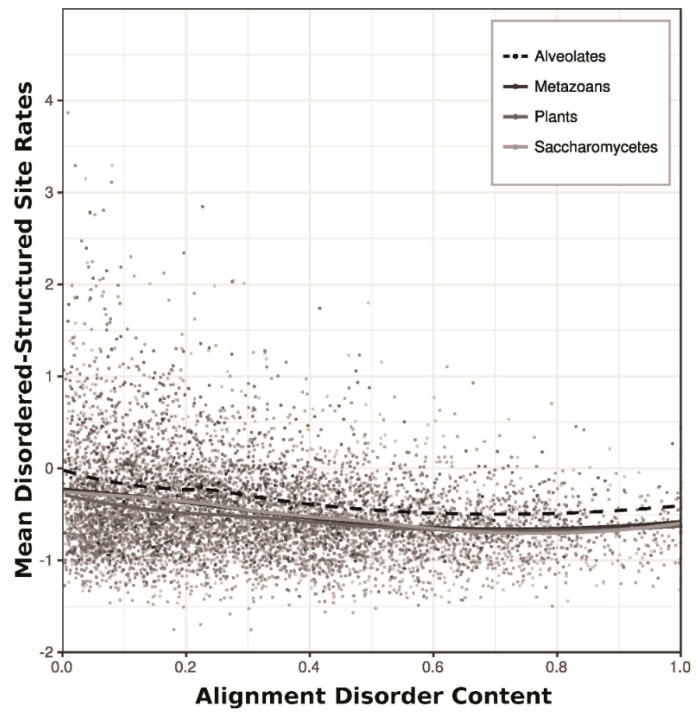
Scatterplot showing the disorder content of clusters (fraction of disordered alignment sites) against the mean rate of sequence evolution among sites predicted to be both disordered and structured. Only sequence clusters containing disordered/structured sites are shown. Trend lines were constructed for each of the four eukaryotic datasets using Loess regression. Note that the Alveolate trend line (dashed) is consistently higher than other lineages.

Loess regression indicated a negative correlation between sequence evolutionary rates of disordered-structured sites and the overall disorder content (fraction of disordered sites) in their respective alignments (Figure 4). This trend is less pronounced in Alveolate alignments than in the other three datasets.

### 3.4. Gene Ontology of Proteins with Disordered-Structured Sites

Analysis of GO (gene ontology) terms in PantherDB [32,33] revealed similar patterns in sequences containing conserved disordered-structured sites within all four eukaryotic lineages. Of the GO annotations found for sequences with conserved disordered-structured sites in *Homo sapiens* (Metazoans), *Arabidopsis thaliana* (Plants), *Saccharomyces cerevisiae* (Saccharomycetes), and *Plasmodium falciparum* (Alveolates), the majority had molecular functions associated with binding (53.3%, 43.0%, 40.5%, and 41.8%, respectively) or catalytic activity (30.8%, 39.5%, 39.6%, and 38.8%, respectively). Additionally, the majority of identified biological processes within these four taxa were either cellular processes (30.1%, 35.3%, 35.4%, and 37.6%, respectively) or metabolic processes (23.9%, 34.9%, 32.1%, and 34.3%, respectively) and the majority of associated cellular components were cell parts (38.9%, 42.0%, 40.2%, and 39.6%, respectively), organelles (30.4%, 32.0%, 30.3%, and 30.8%, respectively) and macromolecular complexes (17.2%, 20.2%, 24.4%, and 24.8%, respectively). In all four taxa, a large fraction of protein classes identified for sequences from alignments with conserved disordered-structured sites were nucleotide-binding proteins (24.3%, 32.1%, 37.5%, and 37.4%, respectively) compared to sequences from alignments lacking conserved disordered-structured sites (11.7%, 15.5%, 17.1%, and 24.8%, respectively). Refer to Supplementary Table S1 for GO term results for sequences with conserved disordered-structured sites from all four representative taxa.

## 4. Discussion

### 4.1. Clustering and Phylogenetics

Previous work by Ahrens et al. [9] highlighted the inherent difficulty of taxon sampling when working with curated molecular datasets—such as the Uniprot Reference Proteome Database [42]—because the bias toward well-studied model organisms is phylogenetically uneven (see Supplementary Figure S1). Indeed, there are large percentages of: (i) Vertebrates in the Metazoan dataset (48%); (ii) Angiosperms (flowering Plants) in the Plant dataset (75%); (iii) *Saccharomyces* congeners in the Saccharomycete dataset (20.5%); and (iv) Plasmodium congeners in the Alveolate dataset (33%). This phylogenetic unevenness can create downstream biases, wherein the sequence clusters suitable for evolutionary analysis primarily depict relationships among well-represented taxa (Vertebrates, Angiosperms, etc.). 

When considering only a single dataset (e.g., Metazoans), it is difficult to determine whether a statistical analysis is biased toward trends in well-represented taxa (e.g., Vertebrates) or truly reflective of more general trends in molecular evolution. By independently analyzing multiple divergent lineages, our statistical results show that there are broad, generally consistent trends across several eukaryotic groups (i.e., in the relationship between structural/functional factors and sequence evolutionary rate) despite the phylogenetic unevenness inherent within the individual datasets.

### 4.2. Structural Prediction

Previous research has revealed that intrinsic disorder is more prevalent in eukaryotic proteins than either Bacteria or Archaea [43,44,45,46]. Rather than simply acting as flexible linkers, some eukaryotic IDR’s occur within functional domains and are crucial to the functions of their associated proteins [47], and many functional IDR’s undergo disorder-to-order transitions in the process of binding to neighboring proteins or nucleotide molecules [48]. Thus, the three factors evaluated in this study (intrinsic disorder, secondary structure, functional domains) appear to be intricately connected and overlapping: intrinsic disorder can occur within functional domains, and transient secondary structures may form within IDR’s to facilitate interactions with other biomolecules. In this light, the combined results of conserved intrinsic disorder, secondary structure and functional domain predictions in an evolutionary context (i.e., multiple sequence alignment sites) appear to be very useful for detecting biologically important sequence regions within proteins.

While sequence-based predictors are not perfectly accurate, our in-silico assignment of three binary states to individual alignment sites (order/disorder, structure/coil, and domain/linker) allowed us to study a wide range of protein alignments from several eukaryotic lineages, including many alignments containing sequences where experimentally-determined structural data is not available. Our analysis workflow (site rate inference, structural prediction, statistical analysis) was applied consistently, such that data arising from different alignments, and different Eukaryote datasets, are directly comparable. Furthermore, by limiting statistical analyses to only gap-free alignment sites with conserved structural predictions, we avoided many error-prone alignment regions as well as inconsistent (and possibly inaccurate) structural assignments. Also, evaluating all combinations of the three binary factors inferred by predictors, we have identified an interesting category of evolutionarily conserved alignment sites (i.e., disordered-structured sites). Notably, such an interplay of structural factors cannot be readily identified via publicly-available experimental data from the Protein Data Bank (PDB) [49], since structural assignments are not provided for regions of intrinsic disorder, where electron density is missing.

### 4.3. Gene Ontology

In prior work on Metazoan protein alignments, Ahrens et al. [9] proposed that disordered-structured sites may be involved in the kinds of disorder-to-order transitions commonly associated with molecular recognition features (MoRFs), wherein the ordered state often adopts secondary structure upon binding to another protein molecule [50,51]. Similar disorder-to-order transitions are important in many nucleic acid binding proteins, especially RNA-binding proteins [52,53,54]. The disorder propensity of these binding regions is thought to confer high specificity, while still allowing binding partners to easily dissociate when necessary [52].

Based on protein class GO terms in our four reference taxa, a large percentage of sequences containing conserved disordered-structured sites are in fact nucleic acid binding proteins (see Supplementary Table S1). Interestingly, a large number of hydrolase proteins also had conserved disordered-structured sites, and there is evidence that some hydrolases rely directly on intrinsic disorder to function. Ubiquitin C-terminal hydrolase activity, for example, is mediated by a disorder-to-order transition within its active site [55,56].

The low amino acid replacement rates we observed in disordered-structured sites suggest selective constraint, likely resulting from the functional importance of transient secondary structure within regions of many eukaryotic proteins [51,52,53,54]. Hence, the joint output of intrinsic disorder and secondary structure predictors in a conserved evolutionary context (i.e., consistent predictions across multiple related sequences) may be useful for identifying protein sites where transitions between disorder and secondary structure are required for protein function.

### 4.4. Intrinsic Disorder in Alveolates

Other researchers have observed that the proteomes of many Alveolate Protists, particularly multi-host pathogens in the clade Apicomplexa, possess a high abundance of proteins with long disordered regions [56,57] and a high fraction of disordered residues in general [46]. Mohan et al. [57] predicted long disordered regions (>30 residues) in most of the protein sequences from the Apicomplexan pathogens *Toxoplasma gondii* (87.8–89.8%) as well as members of the genus *Plasmodium* (75.3–82.5%). Pancsa and Tompa [46] showed that the overall percentage of disordered sites within *T. gondii* proteins was higher than any of the other 193 Eukaryotes they examined, and the disorder percentages of *Plasmodium* spp. proteins were more similar to those of multicellular Eukaryotes (Metazoans, Plants, and Fungi) than other Alveolates. Among the alignment sites containing no gap characters, we observed percentages of conserved disordered sites (6.4–11.6%) that were markedly lower than the overall percentages reported in previous studies [46,57]. Such a disparity is expected, though, since the total number of disordered sites in a given protein sequence exceeds the number of sites with conserved disorder across several related sequences [58]. 

In the case of membrane and secreted proteins, intrinsic disorder in Apicomplexan parasites has a potential dual function: (i) the reduction of antibody binding affinity and (ii) the facilitation of promiscuous attachment to various host cells [59]. Many potential vaccine targets in *Plasmodium* are intrinsically disordered [60], and the erythrocyte binding-like proteins in *P. falciparum* appear to lack transient secondary structures even when recognizing and binding to cell surface receptors during host invasion [61]. Our results indicate that disordered sites in Alveolate proteins also experience higher amino acid replacement rates than other Eukaryotes, and disordered-structured sites in Alveolates are less conserved at the sequence level than in Metazoans, Plants, or Saccharomycetes (Figure 2, Figure 3 and Figure 4). However, recent work has shown that increased rates of protein sequence evolution in disordered regions can result from high positive selection (i.e., an increase in non-synonymous nucleotide substitutions) rather than relaxed purifying selection [62], so the relatively high replacement rates we observed in Alveolate disordered sites may actually be driven by increased pressure for innovation to avoid host recognition and/or to make novel host interactions. Ultimately, these results suggest that developing effective drugs and vaccines targeting Apicomplexan parasites could prove especially difficult, and require a deeper understanding of drug interactions within disordered protein regions.

### 4.5. Statistical Analysis

Across four large-scale molecular datasets, spanning four divergent eukaryotic lineages (Animals, Plants, Fungi, and Protists), we found mostly consistent, statistically significant relationships between three structural/functional factors and site-specific rates of amino acid replacement. By using the equal-probability model from Jukes and Cantor [18] to evaluate rate scores, our results merit a natural, intuitive interpretation—intrinsically disordered sequence sites are more variable than ordered sites, sites in random coils are more variable than sites within secondary structures, and sites in inter-domain linkers are more variable than sites in functional domains. Furthermore, factorial ANOVA indicated widespread confounding interactions among all pairwise combinations of the three factors we tested, as well as significant higher-order interactions beyond what can be observed in trace plots (Figure 3). In fact, the least significant (i.e., highest) *p*-value observed in any factorial ANOVA corresponded to a main effect term (intrinsic disorder in Plants: *p* = 4.13 × 10^−6^), while all other terms across all analyses were highly significant (*p* < 2.2 × 10^−16^). Nonetheless, the first-order interactions appear to follow largely similar patterns in each dataset. One notable exception is the disorder x structure interaction in Alveolates which, although statistically significant, lacks the sign reversal observed in the other three lineages (i.e., disordered-structured sites are more variable on average than ordered, structured sites). Additionally, the disorder x domain interaction seen in Plant sites, where disordered sites within domains tend to be more conserved than ordered domain sites, is less pronounced (but still significant) in the other datasets.

Importantly, the statistical significance of these results (indicated by *p*-values) is consistently high, but the predictive power of the associated factorial models (indicated by correlation coefficients) is consistently low. The residual variance contributing to low model fit can also be seen in the large amount of overlap between the opposing distributions of rate scores (order vs. disorder, structure vs. coil, and domain vs. linker) in every dataset (Figure 2). Hence, it is appropriate to conclude based on our results that ordered sites, for instance, tend to evolve more slowly than disordered sites, but the likelihood that a particular conserved site is ordered is not necessarily high, and said likelihood clearly depends on additional site-specific factors as well (i.e., secondary structure and functional domain involvement). Future large-scale analyses incorporating additional structural factors (e.g., relative solvent exposure) may detect stronger statistical interactions with higher correlations to amino acid replacement rates.

The negative correlation between alignment disorder content (the fraction of disordered sites in an aligned sequence cluster) and the mean relative rate scores of disordered-structured sites within a given alignment suggests that latent structural factors at the sequence level also govern observed rates of amino acid replacement (Figure 4). Such effects are likely nontrivial, considering the unbalanced nature of the site-wise factors discussed here. The prevalence of disordered-structured sites is generally low compared to ordered, structured sites or disordered random coils, and many protein sequences essentially lack intrinsic disorder entirely. Joint analysis of several sequence-level and site-level factors (e.g., via hierarchical linear modelling) may provide deeper insight into the forces driving amino acid replacement.

The complex network of structural and functional properties governing protein (and therefore gene) sequence evolution is a topic of active research [2,63]. To this end, previous work on intrinsic disorder has uncovered similar trends regarding protein sequence conservation [8,9], and much stronger correlations between other protein structural properties and sequence evolutionary rate (e.g., contact number and packing density) have also been observed [2,3,4,64]. Nonetheless, to our knowledge, the results described here represent the most comprehensive evidence for widespread, large-scale structural and functional drivers of eukaryotic sequence evolution to date (Supplementary Figure S1 [65,66]). Furthermore, they reinforce the notion that several factors interact, often in subtle ways, to influence molecular evolution. 

## Figures and Tables

**Table 1:** Dataset-specific information for nonparametric analysis.

Dataset	Metazoans	Plants	Saccharomycetes	Alveolates
Clusters	6938	8266	4494	2697
Sequences	130632	198081	122132	44060
Total Alignment Sites	4677490	4703587	2990109	1640297
Gap-free sites	3217225	2851827	1954761	1179122
Ordered Sites	1819695	1706275	1223656	801629
Disordered Sites	373639	234853	125047	113892
Structured sites	1062380	1014001	722444	417702
Random coil sites	1314563	1064725	670357	424795
Domain sites	1436746	1175745	936813	422813
Linker sites	1368702	1289830	817371	657080
Median Order Rate	−0.599	−0.625	−0.6188	−0.605
Median Disorder Rate	−0.3155	−0.2916	−0.3271	0.1426
Median Structure Rate	−0.5787	−0.6262	−0.5935	−0.605
Median Coil Rate	−0.4682	−0.5013	−0.5603	−0.4542
Median Domain Rate	−0.62345	−0.6679	−0.6353	−0.629
Median Linker Rate	−0.3698	−0.3718	−0.3902	−0.3569

## Supplementary Materials

The following are available online at http://www.mdpi.com/2073-4425/9/11/553/s1, Figure S1: Dataset information, Table S1: GO Term results, Table S2: Nonparametric *Post-Hoc* Multiple Comparison Results.
