# Supplementary material for: Large-Scale Analyses of Site-Specific Evolutionary Rates across Eukaryote Proteomes Reveal Confounding Interactions between Intrinsic Disorder, Secondary Structure, and Functional Domains
Source: Genes (Basel). 2018 Nov 14;9(11):553. doi: 10.3390/genes9110553 (PMC6265720; doi:10.3390/genes9110553)

Supplementary Figure 1 – Dataset information: Cladograms representing evolutionary relationships among the Metazoans, Plants, Saccharomycetes and Alveolates used in this study. Stacked bars (drawn to scale) indicate the fractions of protein sequences from each reference proteome that fall within sequence clusters of various sizes (see individual legends) after clustering analysis. Total proteome sizes (number of sequences in each proteome) are indicated to the right of each stacked bar. Cladograms were drawn according to the NCBI Common Taxonomy Tree [65,66].

Supplementary fig. 1.

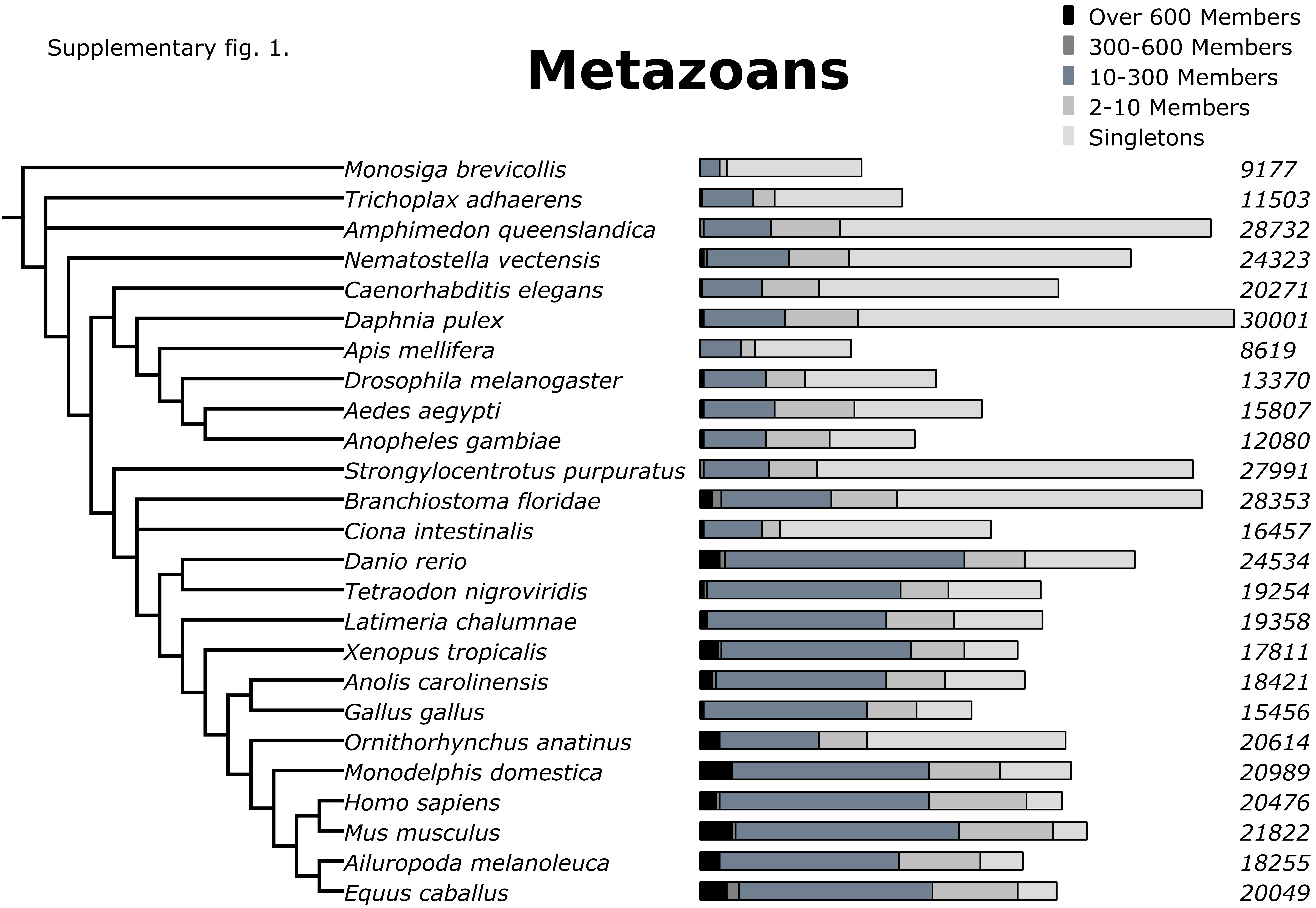

Supplementary fig. 1.

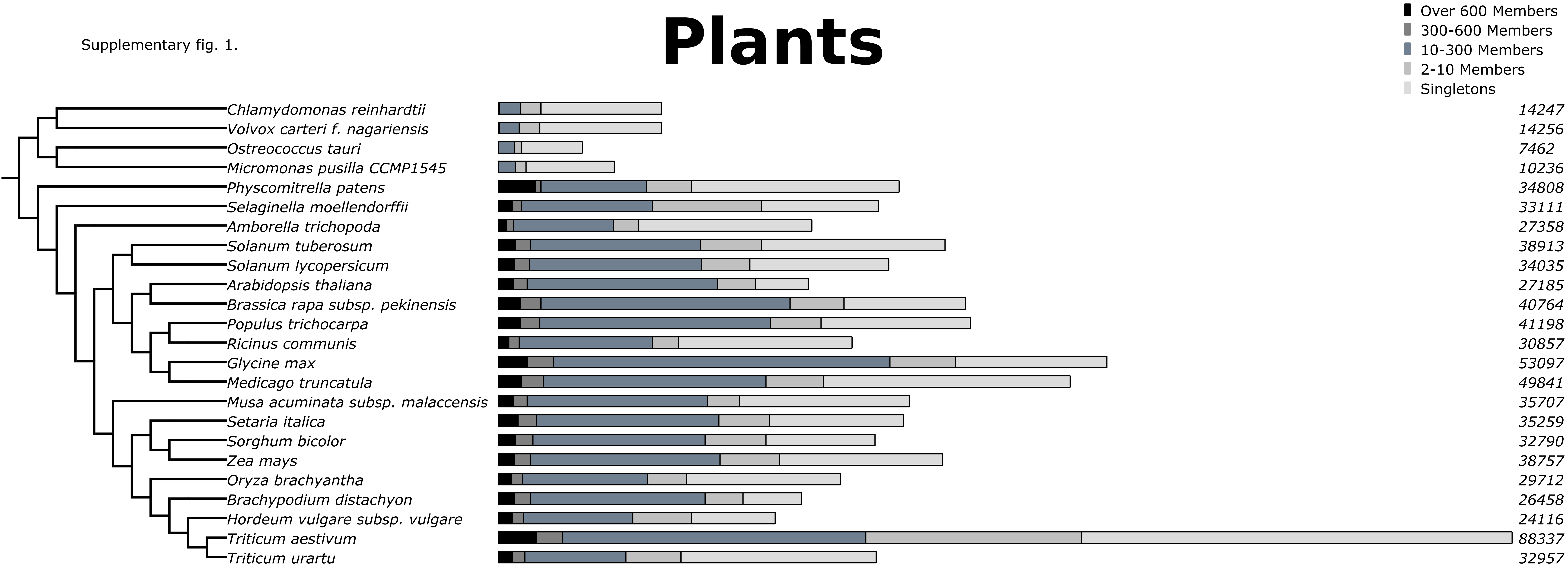

Supplementary fig. 1.

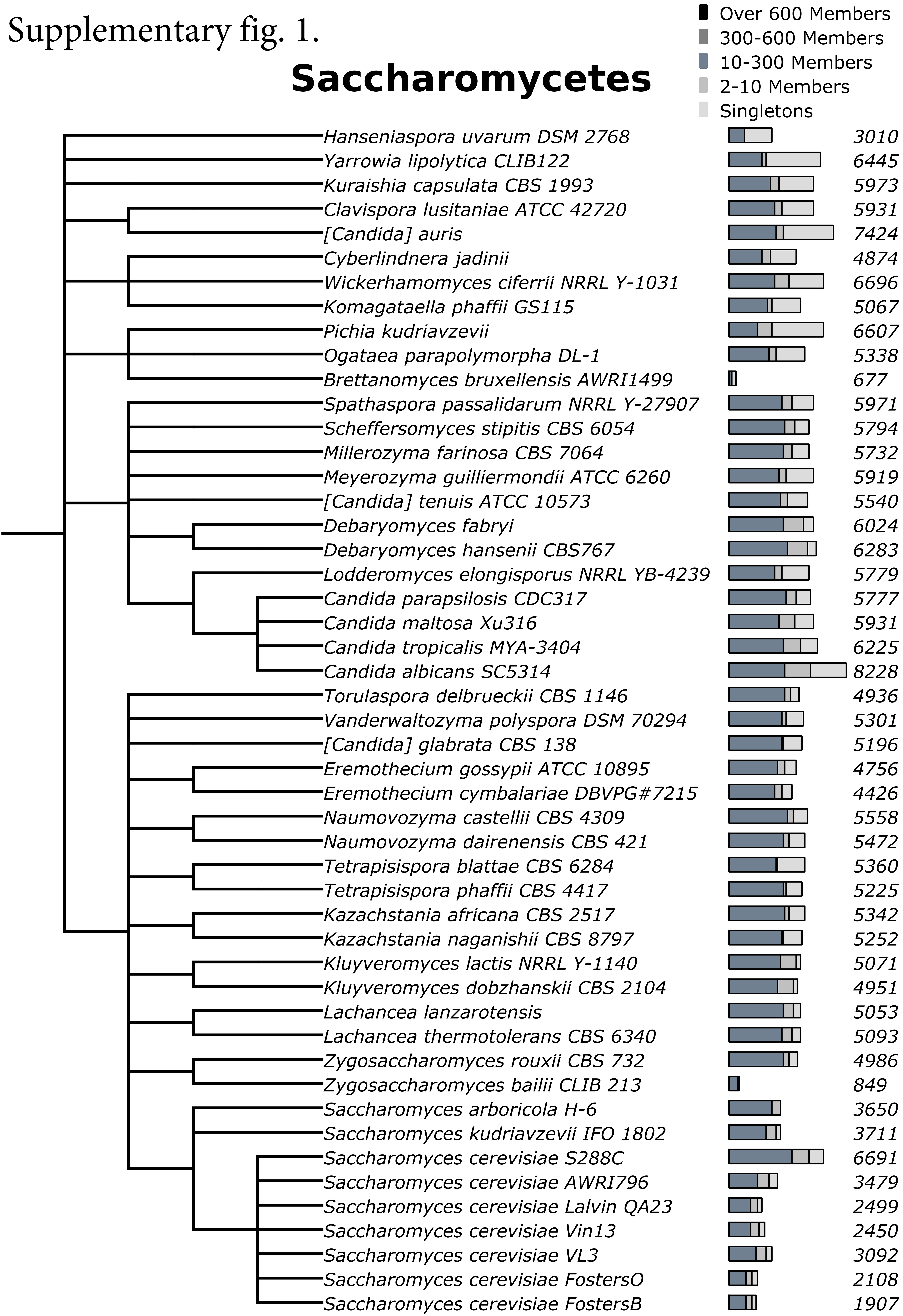

Supplementary fig. 1.

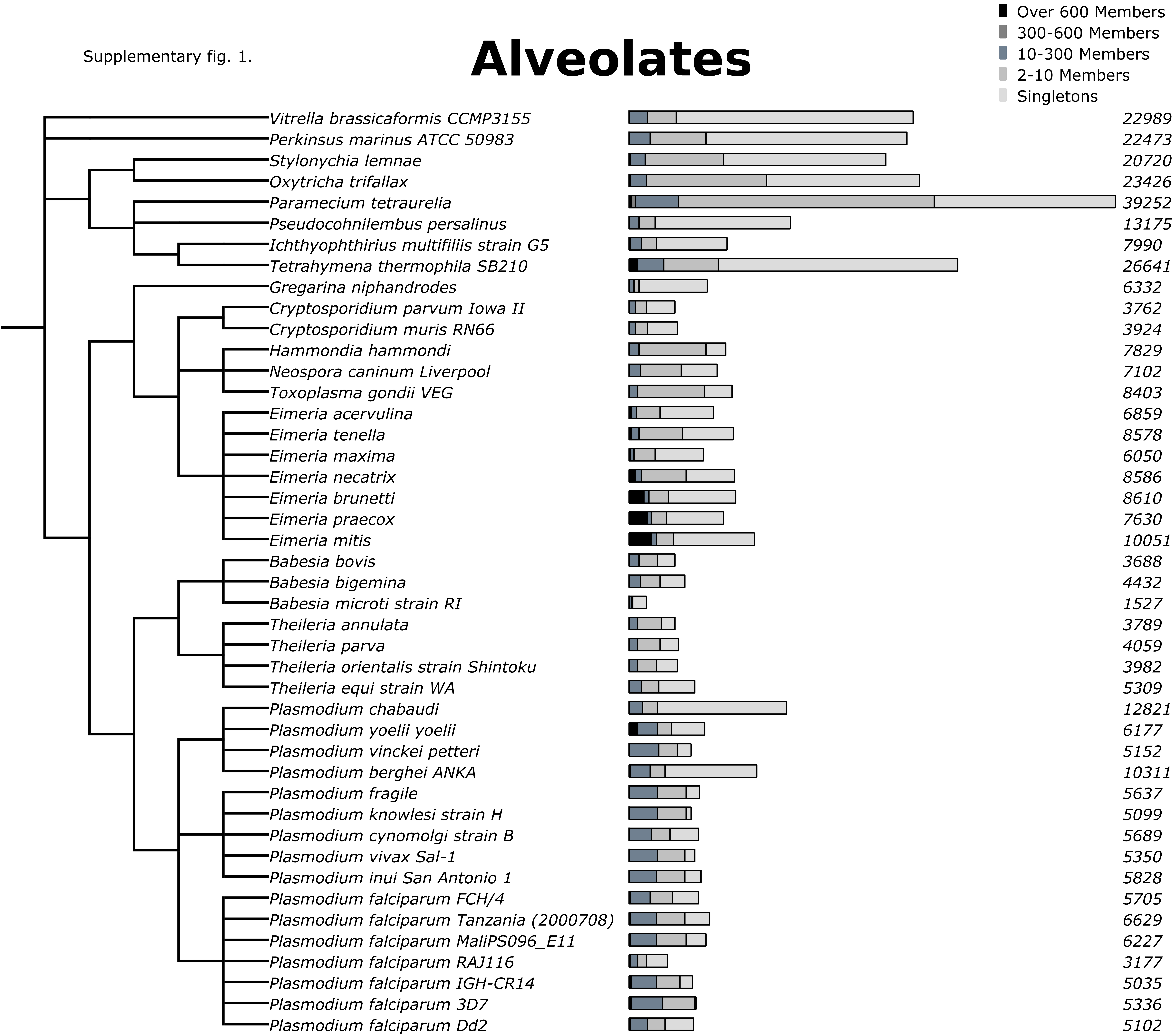

Supplement: Supplementary file 1 [file genes-09-00553-s001.zip › Supplementary_figure_1.pdf]
