# Supplementary material for: Large-Scale Analyses of Site-Specific Evolutionary Rates across Eukaryote Proteomes Reveal Confounding Interactions between Intrinsic Disorder, Secondary Structure, and Functional Domains
Source: Genes (Basel). 2018 Nov 14;9(11):553. doi: 10.3390/genes9110553 (PMC6265720; doi:10.3390/genes9110553)
Supplement: Supplementary file 1 [file genes-09-00553-s001.zip › Supplementary_table_1.pdf]

Supplementary Table 1 – GO Term results: Gene ontology results obtained from PantherDB [32,33] for protein sequences containing conserved disordered-structured sites.

Supplementary table 1.

Molecular Function				
#	GO Term	Count	% Proteins	% Terms
Homo sapiens				
1	binding (GO:0005488)	1127	34.40%	53.30%
2	catalytic activity (GO:0003824)	652	19.90%	30.80%
3	structural molecule activity (GO:000519[illegible]	101	3.10%	4.80%
4	transporter activity (GO:0005215)	97	3.00%	4.60%
5	signal transducer activity (GO:0004871)	56	1.70%	2.60%
6	receptor activity (GO:0004872)	55	1.70%	2.60%
7	translation regulator activity (GO:004518[illegible]	25	0.80%	1.20%
8	channel regulator activity (GO:0016247)	2	0.10%	0.10%
Arabidopsis thaliana				
1	binding (GO:0005488)	574	22.20%	43.00%
2	catalytic activity (GO:0003824)	527	20.40%	39.50%
3	structural molecule activity (GO:000519[illegible]	114	4.40%	8.50%
4	transporter activity (GO:0005215)	44	1.70%	3.30%
5	translation regulator activity (GO:004518[illegible]	38	1.50%	2.80%
6	receptor activity (GO:0004872)	15	0.60%	1.10%
7	signal transducer activity (GO:0004871)	12	0.50%	0.90%
8	antioxidant activity (GO:0016209)	11	0.40%	0.80%
Saccharomyces cerevisiae				
1	binding (GO:0005488)	274	31.50%	40.50%
2	catalytic activity (GO:0003824)	268	30.80%	39.60%
3	structural molecule activity (GO:000519[illegible]	78	9.00%	11.50%
4	transporter activity (GO:0005215)	26	3.00%	3.80%
5	translation regulator activity (GO:004518[illegible]	23	2.60%	3.40%
6	signal transducer activity (GO:0004871)	4	0.50%	0.60%
7	receptor activity (GO:0004872)	3	0.30%	0.40%
8	antioxidant activity (GO:0016209)	1	0.10%	0.10%
Plasmodium falciparum				
1	binding (GO:0005488)	157	29.50%	41.80%
2	catalytic activity (GO:0003824)	146	27.40%	38.80%
3	structural molecule activity (GO:000519[illegible]	36	6.80%	9.60%
4	transporter activity (GO:0005215)	18	3.40%	4.80%
5	translation regulator activity (GO:004518[illegible]	17	3.20%	4.50%
6	receptor activity (GO:0004872)	1	0.20%	0.30%
7	antioxidant activity (GO:0016209)	1	0.20%	0.30%

Supplementary table 1.

Biological Process				
#	GO Term	Count	% Proteins	% Terms
Homo sapiens				
1	cellular process (GO:0009987)	1533	46.80%	30.10%
2	metabolic process (GO:0008152)	1218	37.20%	23.90%
3	cellular component organization or biogenesis (GO:0071840)	529	16.20%	10.40%
4	biological regulation (GO:0065007)	369	11.30%	7.20%
5	developmental process (GO:0032502)	362	11.10%	7.10%
6	localization (GO:0051179)	329	10.00%	6.50%
7	response to stimulus (GO:0050896)	314	9.60%	6.20%
8	multicellular organismal process (GO:0032501)	244	7.50%	4.80%
9	immune system process (GO:0002376)	64	2.00%	1.30%
10	biological adhesion (GO:0022610)	54	1.60%	1.10%
11	locomotion (GO:0040011)	42	1.30%	0.80%
12	reproduction (GO:0000003)	31	0.90%	0.60%
13	rhythmic process (GO:0048511)	5	0.20%	0.10%
14	growth (GO:0040007)	4	0.10%	0.10%
Arabidopsis thaliana				
1	metabolic process (GO:0008152)	903	34.90%	35.30%
2	cellular process (GO:0009987)	894	34.50%	34.90%
3	cellular component organization or biogenesis (GO:0071840)	294	11.40%	11.50%
4	localization (GO:0051179)	164	6.30%	6.40%
5	response to stimulus (GO:0050896)	134	5.20%	5.20%
6	biological regulation (GO:0065007)	104	4.00%	4.10%
7	developmental process (GO:0032502)	32	1.20%	1.30%
8	reproduction (GO:0000003)	20	0.80%	0.80%
9	multicellular organismal process (GO:0032501)	11	0.40%	0.40%
10	rhythmic process (GO:0048511)	2	0.10%	0.10%
Saccharomyces cerevisiae				
1	cellular process (GO:0009987)	478	54.90%	35.40%
2	metabolic process (GO:0008152)	434	49.80%	32.10%
3	cellular component organization or biogenesis (GO:0071840)	226	25.90%	16.70%
4	localization (GO:0051179)	95	10.90%	7.00%
5	response to stimulus (GO:0050896)	59	6.80%	4.40%
6	biological regulation (GO:0065007)	55	6.30%	4.10%
7	reproduction (GO:0000003)	4	0.50%	0.30%
8	developmental process (GO:0032502)	1	0.10%	0.10%
Plasmodium falciparum				
1	cellular process (GO:0009987)	275	51.60%	37.60%
2	metabolic process (GO:0008152)	251	47.10%	34.30%

Supplementary table 1.

3	cellular component organization or biogenesis (GO:0071840)	103	19.30%	14.10%
4	localization (GO:0051179)	54	10.10%	7.40%
5	response to stimulus (GO:0050896)	24	4.50%	3.30%
6	biological regulation (GO:0065007)	17	3.20%	2.30%
7	reproduction (GO:0000003)	4	0.80%	0.50%
8	locomotion (GO:0040011)	2	0.40%	0.30%
9	developmental process (GO:0032502)	1	0.20%	0.10%

Supplementary table 1.

Cellular Component				
#	GO Term	Count	% Proteins	% Terms
Homo sapiens				
1	cell part (GO:0044464)	1248	38.10%	38.90%
2	organelle (GO:0043226)	976	29.80%	30.40%
3	macromolecular complex (GO:0032991	553	16.90%	17.20%
4	membrane (GO:0016020)	295	9.00%	9.20%
5	extracellular region (GO:0005576)	60	1.80%	1.90%
6	cell junction (GO:0030054)	31	0.90%	1.00%
7	synapse (GO:0045202)	29	0.90%	0.90%
8	extracellular matrix (GO:0031012)	16	0.50%	0.50%
Arabidopsis thaliana				
1	cell part (GO:0044464)	936	36.20%	42.00%
2	organelle (GO:0043226)	712	27.50%	32.00%
3	macromolecular complex (GO:0032991	450	17.40%	20.20%
4	membrane (GO:0016020)	118	4.60%	5.30%
5	extracellular region (GO:0005576)	9	0.30%	0.40%
6	nucleoid (GO:0009295)	1	0.00%	0.00%
7	cell junction (GO:0030054)	1	0.00%	0.00%
Saccharomyces cerevisiae				
1	cell part (GO:0044464)	484	55.60%	40.20%
2	organelle (GO:0043226)	365	41.90%	30.30%
3	macromolecular complex (GO:0032991	294	33.80%	24.40%
4	membrane (GO:0016020)	61	7.00%	5.10%
5	extracellular region (GO:0005576)	1	0.10%	0.10%
Plasmodium falciparum				
1	cell part (GO:0044464)	243	45.60%	39.60%
2	organelle (GO:0043226)	189	35.50%	30.80%
3	macromolecular complex (GO:0032991	152	28.50%	24.80%
4	membrane (GO:0016020)	27	5.10%	4.40%
5	extracellular region (GO:0005576)	3	0.60%	0.50%

Supplementary table 1.

Protein Class				
#	GO Term	Count	% Proteins	% Terms
Homo sapiens				
1	nucleic acid binding (PC00171)	529	16.20%	24.30%
2	transcription factor (PC00218)	367	11.20%	16.90%
3	hydrolase (PC00121)	209	6.40%	9.60%
4	enzyme modulator (PC00095)	178	5.40%	8.20%
5	cytoskeletal protein (PC00085)	129	3.90%	5.90%
6	transferase (PC00220)	122	3.70%	5.60%
7	membrane traffic protein (PC00150)	87	2.70%	4.00%
8	signaling molecule (PC00207)	79	2.40%	3.60%
9	transporter (PC00227)	55	1.70%	2.50%
10	receptor (PC00197)	53	1.60%	2.40%
11	ligase (PC00142)	47	1.40%	2.20%
12	transfer/carrier protein (PC00219)	45	1.40%	2.10%
13	calcium-binding protein (PC00060)	40	1.20%	1.80%
14	chaperone (PC00072)	31	0.90%	1.40%
15	oxidoreductase (PC00176)	29	0.90%	1.30%
16	cell adhesion molecule (PC00069)	29	0.90%	1.30%
17	transmembrane receptor regulatory/adaptor protein (PC002	27	0.80%	1.20%
18	defense/immunity protein (PC00090)	26	0.80%	1.20%
19	cell junction protein (PC00070)	26	0.80%	1.20%
20	extracellular matrix protein (PC00102)	22	0.70%	1.00%
21	structural protein (PC00211)	16	0.50%	0.70%
22	isomerase (PC00135)	15	0.50%	0.70%
23	lyase (PC00144)	11	0.30%	0.50%
24	viral protein (PC00237)	1	0.00%	0.00%
25	surfactant (PC00212)	1	0.00%	0.00%
Arabidopsis thaliana				
1	nucleic acid binding (PC00171)	403	15.60%	32.10%
2	hydrolase (PC00121)	175	6.80%	13.90%
3	transferase (PC00220)	112	4.30%	8.90%
4	transcription factor (PC00218)	97	3.70%	7.70%
5	oxidoreductase (PC00176)	64	2.50%	5.10%
6	enzyme modulator (PC00095)	63	2.40%	5.00%
7	cytoskeletal protein (PC00085)	56	2.20%	4.50%
8	transporter (PC00227)	45	1.70%	3.60%
9	membrane traffic protein (PC00150)	41	1.60%	3.30%
10	lyase (PC00144)	39	1.50%	3.10%
11	ligase (PC00142)	36	1.40%	2.90%

Supplementary table 1.

12	isomerase (PC00135)	30	1.20%	2.40%
13	chaperone (PC00072)	22	0.90%	1.80%
14	transfer/carrier protein (PC00219)	19	0.70%	1.50%
15	calcium-binding protein (PC00060)	17	0.70%	1.40%
16	defense/immunity protein (PC00090)	7	0.30%	0.60%
17	signaling molecule (PC00207)	6	0.20%	0.50%
18	structural protein (PC00211)	6	0.20%	0.50%
19	receptor (PC00197)	6	0.20%	0.50%
20	extracellular matrix protein (PC00102)	3	0.10%	0.20%
21	transmembrane receptor regulatory/adaptor protein (PC002	3	0.10%	0.20%
22	cell adhesion molecule (PC00069)	3	0.10%	0.20%
23	storage protein (PC00210)	2	0.10%	0.20%
Saccharomyces cerevisiae				
1	nucleic acid binding (PC00171)	227	26.10%	37.50%
2	hydrolase (PC00121)	68	7.80%	11.20%
3	transferase (PC00220)	50	5.70%	8.30%
4	enzyme modulator (PC00095)	44	5.10%	7.30%
5	transcription factor (PC00218)	39	4.50%	6.40%
6	transporter (PC00227)	25	2.90%	4.10%
7	ligase (PC00142)	23	2.60%	3.80%
8	membrane traffic protein (PC00150)	22	2.50%	3.60%
9	cytoskeletal protein (PC00085)	20	2.30%	3.30%
10	oxidoreductase (PC00176)	20	2.30%	3.30%
11	lyase (PC00144)	14	1.60%	2.30%
12	transfer/carrier protein (PC00219)	13	1.50%	2.10%
13	chaperone (PC00072)	10	1.10%	1.70%
14	calcium-binding protein (PC00060)	7	0.80%	1.20%
15	isomerase (PC00135)	7	0.80%	1.20%
16	signaling molecule (PC00207)	5	0.60%	0.80%
17	cell junction protein (PC00070)	4	0.50%	0.70%
18	receptor (PC00197)	4	0.50%	0.70%
19	defense/immunity protein (PC00090)	1	0.10%	0.20%
20	structural protein (PC00211)	1	0.10%	0.20%
21	storage protein (PC00210)	1	0.10%	0.20%
Plasmodium falciparum				
1	nucleic acid binding (PC00171)	143	26.80%	37.40%
2	hydrolase (PC00121)	48	9.00%	12.60%
3	enzyme modulator (PC00095)	34	6.40%	8.90%
4	transferase (PC00220)	28	5.30%	7.30%
5	cytoskeletal protein (PC00085)	18	3.40%	4.70%

Supplementary table 1.

6	ligase (PC00142)	17	3.20%	4.50%
7	membrane traffic protein (PC00150)	15	2.80%	3.90%
8	transporter (PC00227)	14	2.60%	3.70%
9	transcription factor (PC00218)	13	2.40%	3.40%
10	calcium-binding protein (PC00060)	8	1.50%	2.10%
11	transfer/carrier protein (PC00219)	8	1.50%	2.10%
12	chaperone (PC00072)	8	1.50%	2.10%
13	isomerase (PC00135)	8	1.50%	2.10%
14	oxidoreductase (PC00176)	5	0.90%	1.30%
15	lyase (PC00144)	3	0.60%	0.80%
16	extracellular matrix protein (PC00102)	2	0.40%	0.50%
17	signaling molecule (PC00207)	2	0.40%	0.50%
18	cell junction protein (PC00070)	2	0.40%	0.50%
19	structural protein (PC00211)	2	0.40%	0.50%
20	receptor (PC00197)	2	0.40%	0.50%
21	transmembrane receptor regulatory/adaptor protein (PC002	1	0.20%	0.30%
22	defense/immunity protein (PC00090)	1	0.20%	0.30%

Supplementary table 1.

#	GO Term	Count	% Proteins	% Term
	Pathway			
Homo sapiens				
1	Wnt signaling pathway (P00057)	75	2.30%	6.90%
2	Gonadotropin-releasing hormone receptor pathway (P06664)	64	2.00%	5.90%
3	Integrin signalling pathway (P00034)	40	1.20%	3.70%
4	PDGF signaling pathway (P00047)	38	1.20%	3.50%
5	CCKR signaling map (P06959)	37	1.10%	3.40%
6	Inflammation mediated by chemokine and cytokine signaling pathway (P00031)	36	1.10%	3.30%
7	Angiogenesis (P00005)	35	1.10%	3.20%
8	Alzheimer disease-presenilin pathway (P00004)	30	0.90%	2.80%
9	Cadherin signaling pathway (P00012)	30	0.90%	2.80%
10	Huntington disease (P00029)	26	0.80%	2.40%
11	FGF signaling pathway (P00021)	25	0.80%	2.30%
12	EGF receptor signaling pathway (P00018)	25	0.80%	2.30%
13	Apoptosis signaling pathway (P00006)	23	0.70%	2.10%
14	Transcription regulation by bZIP transcription factor (P00055)	20	0.60%	1.90%
15	TGF-beta signaling pathway (P00052)	20	0.60%	1.90%
16	Heterotrimeric G-protein signaling pathway-Gq alpha and Go alpha mediated pathway (P(	18	0.50%	1.70%
17	T cell activation (P00053)	17	0.50%	1.60%
18	Cytoskeletal regulation by Rho GTPase (P00016)	17	0.50%	1.60%
19	p53 pathway (P00059)	16	0.50%	1.50%
20	Parkinson disease (P00049)	16	0.50%	1.50%
21	General transcription regulation (P00023)	15	0.50%	1.40%
22	Endothelin signaling pathway (P00019)	15	0.50%	1.40%
23	B cell activation (P00010)	15	0.50%	1.40%
24	VEGF signaling pathway (P00056)	14	0.40%	1.30%
25	Interleukin signaling pathway (P00036)	14	0.40%	1.30%
26	Alzheimer disease-amyloid secretase pathway (P00003)	13	0.40%	1.20%
27	p53 pathway feedback loops 2 (P04398)	13	0.40%	1.20%
28	Heterotrimeric G-protein signaling pathway-Gi alpha and Gs alpha mediated pathway (P0(	13	0.40%	1.20%
29	Toll receptor signaling pathway (P00054)	12	0.40%	1.10%
30	Nicotinic acetylcholine receptor signaling pathway (P00044)	12	0.40%	1.10%
31	Ras Pathway (P04393)	11	0.30%	1.00%
32	Muscarinic acetylcholine receptor 1 and 3 signaling pathway (P00042)	10	0.30%	0.90%
33	Insulin/IGF pathway-protein kinase B signaling cascade (P00033)	10	0.30%	0.90%
34	Axon guidance mediated by netrin (P00009)	9	0.30%	0.80%
35	Ubiquitin proteasome pathway (P00060)	9	0.30%	0.80%
36	Hypoxia response via HIF activation (P00030)	9	0.30%	0.80%
37	5HT2 type receptor mediated signaling pathway (P04374)	9	0.30%	0.80%

Supplementary table 1.

38	Oxidative stress response (P00046)	8	0.20%	0.70%
39	Notch signaling pathway (P00045)	8	0.20%	0.70%
40	Thyrotropin-releasing hormone receptor signaling pathway (P04394)	8	0.20%	0.70%
41	p38 MAPK pathway (P05918)	8	0.20%	0.70%
42	DNA replication (P00017)	8	0.20%	0.70%
43	Synaptic vesicle trafficking (P05734)	7	0.20%	0.60%
44	Ionotropic glutamate receptor pathway (P00037)	7	0.20%	0.60%
45	Interferon-gamma signaling pathway (P00035)	7	0.20%	0.60%
46	Oxytocin receptor mediated signaling pathway (P04391)	7	0.20%	0.60%
47	Histamine H1 receptor mediated signaling pathway (P04385)	7	0.20%	0.60%
48	PI3 kinase pathway (P00048)	6	0.20%	0.60%
49	Hedgehog signaling pathway (P00025)	6	0.20%	0.60%
50	Angiotensin II-stimulated signaling through G proteins and beta-arrestin (P05911)	6	0.20%	0.60%
51	Circadian clock system (P00015)	6	0.20%	0.60%
52	Cell cycle (P00013)	6	0.20%	0.60%
53	Axon guidance mediated by Slit/Robo (P00008)	5	0.20%	0.50%
54	Alpha adrenergic receptor signaling pathway (P00002)	5	0.20%	0.50%
55	mRNA splicing (P00058)	5	0.20%	0.50%
56	GABA-B receptor II signaling (P05731)	5	0.20%	0.50%
57	Insulin/IGF pathway-mitogen activated protein kinase kinase/MAP kinase cascade (P0003	5	0.20%	0.50%
58	Nicotine pharmacodynamics pathway (P06587)	5	0.20%	0.50%
59	Blood coagulation (P00011)	5	0.20%	0.50%
60	Beta2 adrenergic receptor signaling pathway (P04378)	5	0.20%	0.50%
61	Beta1 adrenergic receptor signaling pathway (P04377)	5	0.20%	0.50%
62	TCA cycle (P00051)	4	0.10%	0.40%
63	De novo pyrimidine ribonucleotides biosythesis (P02740)	4	0.10%	0.40%
64	Metabotropic glutamate receptor group II pathway (P00040)	4	0.10%	0.40%
65	Metabotropic glutamate receptor group III pathway (P00039)	4	0.10%	0.40%
66	Vasopressin synthesis (P04395)	4	0.10%	0.40%
67	P53 pathway feedback loops 1 (P04392)	4	0.10%	0.40%
68	Opioid proopiomelanocortin pathway (P05917)	4	0.10%	0.40%
69	Opioid proenkephalin pathway (P05915)	4	0.10%	0.40%
70	Dopamine receptor mediated signaling pathway (P05912)	4	0.10%	0.40%
71	Adrenaline and noradrenaline biosynthesis (P00001)	3	0.10%	0.30%
72	Heme biosynthesis (P02746)	3	0.10%	0.30%
73	Plasminogen activating cascade (P00050)	3	0.10%	0.30%
74	Muscarinic acetylcholine receptor 2 and 4 signaling pathway (P00043)	3	0.10%	0.30%
75	Metabotropic glutamate receptor group I pathway (P00041)	3	0.10%	0.30%
76	Endogenous cannabinoid signaling (P05730)	3	0.10%	0.30%
77	JAK/STAT signaling pathway (P00038)	3	0.10%	0.30%

Supplementary table 1.

78	p53 pathway by glucose deprivation (P04397)	3	0.10%	0.30%
79	General transcription by RNA polymerase I (P00022)	3	0.10%	0.30%
80	Pyruvate metabolism (P02772)	3	0.10%	0.30%
81	Cortocotropin releasing factor receptor signaling pathway (P04380)	3	0.10%	0.30%
82	5HT4 type receptor mediated signaling pathway (P04376)	3	0.10%	0.30%
83	5HT3 type receptor mediated signaling pathway (P04375)	3	0.10%	0.30%
84	5HT1 type receptor mediated signaling pathway (P04373)	3	0.10%	0.30%
85	Axon guidance mediated by semaphorins (P00007)	2	0.10%	0.20%
86	Methylmalonyl pathway (P02755)	2	0.10%	0.20%
87	Isoleucine biosynthesis (P02748)	2	0.10%	0.20%
88	De novo purine biosynthesis (P02738)	2	0.10%	0.20%
89	ATP synthesis (P02721)	2	0.10%	0.20%
90	Vitamin D metabolism and pathway (P04396)	2	0.10%	0.20%
91	Glycolysis (P00024)	2	0.10%	0.20%
92	Succinate to proprionate conversion (P02777)	2	0.10%	0.20%
93	FAS signaling pathway (P00020)	2	0.10%	0.20%
94	5-Hydroxytryptamine degredation (P04372)	2	0.10%	0.20%
95	SCW signaling pathway (P06216)	1	0.00%	0.10%
96	GBB signaling pathway (P06214)	1	0.00%	0.10%
97	DPP signaling pathway (P06213)	1	0.00%	0.10%
98	DPP-SCW signaling pathway (P06212)	1	0.00%	0.10%
99	BMP/activin signaling pathway-drosophila (P06211)	1	0.00%	0.10%
100	Pyridoxal-5-phosphate biosynthesis (P02759)	1	0.00%	0.10%
101	Mannose metabolism (P02752)	1	0.00%	0.10%
102	Lipoate_biosynthesis (P02750)	1	0.00%	0.10%
103	Fructose galactose metabolism (P02744)	1	0.00%	0.10%
104	De novo pyrimidine deoxyribonucleotide biosynthesis (P02739)	1	0.00%	0.10%
105	Cysteine biosynthesis (P02737)	1	0.00%	0.10%
106	Arginine biosynthesis (P02728)	1	0.00%	0.10%
107	Vitamin B6 metabolism (P02787)	1	0.00%	0.10%
108	Valine biosynthesis (P02785)	1	0.00%	0.10%
109	Anandamide degradation (P05728)	1	0.00%	0.10%
110	Opioid prodynorphin pathway (P05916)	1	0.00%	0.10%
111	Salvage pyrimidine ribonucleotides (P02775)	1	0.00%	0.10%
112	Enkephalin release (P05913)	1	0.00%	0.10%
113	Pyridoxal phosphate salvage pathway (P02770)	1	0.00%	0.10%
114	Proline biosynthesis (P02768)	1	0.00%	0.10%
115	Beta3 adrenergic receptor signaling pathway (P04379)	1	0.00%	0.10%
116	Pentose phosphate pathway (P02762)	1	0.00%	0.10%
Arabidopsis thaliana				

Supplementary table 1.

1	General transcription regulation (P00023)	17	0.70%	5.50%
2	Transcription regulation by bZIP transcription factor (P00055)	16	0.60%	5.20%
3	Ubiquitin proteasome pathway (P00060)	10	0.40%	3.30%
4	Huntington disease (P00029)	9	0.30%	2.90%
5	PDGF signaling pathway (P00047)	8	0.30%	2.60%
6	DNA replication (P00017)	8	0.30%	2.60%
7	Wnt signaling pathway (P00057)	7	0.30%	2.30%
8	Parkinson disease (P00049)	7	0.30%	2.30%
9	De novo purine biosynthesis (P02738)	7	0.30%	2.30%
10	Nicotinic acetylcholine receptor signaling pathway (P00044)	7	0.30%	2.30%
11	Ras Pathway (P04393)	7	0.30%	2.30%
12	EGF receptor signaling pathway (P00018)	7	0.30%	2.30%
13	Adrenaline and noradrenaline biosynthesis (P00001)	6	0.20%	2.00%
14	Histidine biosynthesis (P02747)	6	0.20%	2.00%
15	Heme biosynthesis (P02746)	6	0.20%	2.00%
16	Muscarinic acetylcholine receptor 2 and 4 signaling pathway (P00043)	6	0.20%	2.00%
17	Insulin/IGF pathway-mitogen activated protein kinase kinase/MAP kinase cascade (P0003	6	0.20%	2.00%
18	Gonadotropin-releasing hormone receptor pathway (P06664)	5	0.20%	1.60%
19	Interleukin signaling pathway (P00036)	5	0.20%	1.60%
20	Inflammation mediated by chemokine and cytokine signaling pathway (P00031)	5	0.20%	1.60%
21	TCA cycle (P00051)	4	0.20%	1.30%
22	Oxidative stress response (P00046)	4	0.20%	1.30%
23	Ionotropic glutamate receptor pathway (P00037)	4	0.20%	1.30%
24	Tryptophan biosynthesis (P02783)	4	0.20%	1.30%
25	FGF signaling pathway (P00021)	4	0.20%	1.30%
26	FAS signaling pathway (P00020)	4	0.20%	1.30%
27	S-adenosylmethionine biosynthesis (P02773)	4	0.20%	1.30%
28	p53 pathway (P00059)	3	0.10%	1.00%
29	De novo pyrimidine ribonucleotides biosythesis (P02740)	3	0.10%	1.00%
30	Muscarinic acetylcholine receptor 1 and 3 signaling pathway (P00042)	3	0.10%	1.00%
31	Metabotropic glutamate receptor group II pathway (P00040)	3	0.10%	1.00%
32	Arginine biosynthesis (P02728)	3	0.10%	1.00%
33	Metabotropic glutamate receptor group III pathway (P00039)	3	0.10%	1.00%
34	Integrin signalling pathway (P00034)	3	0.10%	1.00%
35	Thyrotropin-releasing hormone receptor signaling pathway (P04394)	3	0.10%	1.00%
36	Oxytocin receptor mediated signaling pathway (P04391)	3	0.10%	1.00%
37	p38 MAPK pathway (P05918)	3	0.10%	1.00%
38	Opioid proopiomelanocortin pathway (P05917)	3	0.10%	1.00%
39	Opioid prodynorphin pathway (P05916)	3	0.10%	1.00%
40	Opioid proenkephalin pathway (P05915)	3	0.10%	1.00%

Supplementary table 1.

41	Dopamine receptor mediated signaling pathway (P05912)	3	0.10%	1.00%
42	Pyruvate metabolism (P02772)	3	0.10%	1.00%
43	Cortocotropin releasing factor receptor signaling pathway (P04380)	3	0.10%	1.00%
44	Beta3 adrenergic receptor signaling pathway (P04379)	3	0.10%	1.00%
45	Beta2 adrenergic receptor signaling pathway (P04378)	3	0.10%	1.00%
46	Beta1 adrenergic receptor signaling pathway (P04377)	3	0.10%	1.00%
47	5HT4 type receptor mediated signaling pathway (P04376)	3	0.10%	1.00%
48	5HT3 type receptor mediated signaling pathway (P04375)	3	0.10%	1.00%
49	5HT2 type receptor mediated signaling pathway (P04374)	3	0.10%	1.00%
50	5HT1 type receptor mediated signaling pathway (P04373)	3	0.10%	1.00%
51	Apoptosis signaling pathway (P00006)	2	0.10%	0.70%
52	O-antigen biosynthesis (P02757)	2	0.10%	0.70%
53	Methionine biosynthesis (P02753)	2	0.10%	0.70%
54	CCKR signaling map (P06959)	2	0.10%	0.70%
55	Lysine biosynthesis (P02751)	2	0.10%	0.70%
56	Fructose galactose metabolism (P02744)	2	0.10%	0.70%
57	TGF-beta signaling pathway (P00052)	2	0.10%	0.70%
58	PI3 kinase pathway (P00048)	2	0.10%	0.70%
59	Adenine and hypoxanthine salvage pathway (P02723)	2	0.10%	0.70%
60	Insulin/IGF pathway-protein kinase B signaling cascade (P00033)	2	0.10%	0.70%
61	p53 pathway feedback loops 2 (P04398)	2	0.10%	0.70%
62	Vitamin D metabolism and pathway (P04396)	2	0.10%	0.70%
63	General transcription by RNA polymerase I (P00022)	2	0.10%	0.70%
64	Pentose phosphate pathway (P02762)	2	0.10%	0.70%
65	Pyridoxal-5-phosphate biosynthesis (P02759)	1	0.00%	0.30%
66	N-acetylglucosamine metabolism (P02756)	1	0.00%	0.30%
67	Methylmalonyl pathway (P02755)	1	0.00%	0.30%
68	Isoleucine biosynthesis (P02748)	1	0.00%	0.30%
69	mRNA splicing (P00058)	1	0.00%	0.30%
70	Glutamine glutamate conversion (P02745)	1	0.00%	0.30%
71	Notch signaling pathway (P00045)	1	0.00%	0.30%
72	Chorismate biosynthesis (P02734)	1	0.00%	0.30%
73	Biotin biosynthesis (P02731)	1	0.00%	0.30%
74	Synaptic vesicle trafficking (P05734)	1	0.00%	0.30%
75	Ascorbate degradation (P02729)	1	0.00%	0.30%
76	Allantoin degradation (P02725)	1	0.00%	0.30%
77	Interferon-gamma signaling pathway (P00035)	1	0.00%	0.30%
78	Vitamin B6 metabolism (P02787)	1	0.00%	0.30%
79	Valine biosynthesis (P02785)	1	0.00%	0.30%
80	Hypoxia response via HIF activation (P00030)	1	0.00%	0.30%

Supplementary table 1.

81	Hedgehog signaling pathway (P00025)	1	0.00%	0.30%
82	Succinate to proprionate conversion (P02777)	1	0.00%	0.30%
83	Serine glycine biosynthesis (P02776)	1	0.00%	0.30%
84	Salvage pyrimidine ribonucleotides (P02775)	1	0.00%	0.30%
85	Pyrimidine Metabolism (P02771)	1	0.00%	0.30%
86	Pyridoxal phosphate salvage pathway (P02770)	1	0.00%	0.30%
87	Circadian clock system (P00015)	1	0.00%	0.30%
88	Cell cycle (P00013)	1	0.00%	0.30%
89	Phenylethylamine degradation (P02766)	1	0.00%	0.30%
90	Peptidoglycan biosynthesis (P02763)	1	0.00%	0.30%
91	5-Hydroxytryptamine degredation (P04372)	1	0.00%	0.30%
Saccharomyces cerevisiae				
1	Transcription regulation by bZIP transcription factor (P00055)	16	1.80%	8.00%
2	General transcription regulation (P00023)	15	1.70%	7.50%
3	Parkinson disease (P00049)	14	1.60%	7.00%
4	Ubiquitin proteasome pathway (P00060)	13	1.50%	6.50%
5	Apoptosis signaling pathway (P00006)	10	1.10%	5.00%
6	Wnt signaling pathway (P00057)	10	1.10%	5.00%
7	Nicotinic acetylcholine receptor signaling pathway (P00044)	7	0.80%	3.50%
8	Glycolysis (P00024)	6	0.70%	3.00%
9	De novo purine biosynthesis (P02738)	5	0.60%	2.50%
10	TCA cycle (P00051)	4	0.50%	2.00%
11	EGF receptor signaling pathway (P00018)	4	0.50%	2.00%
12	DNA replication (P00017)	4	0.50%	2.00%
13	Isoleucine biosynthesis (P02748)	3	0.30%	1.50%
14	Heme biosynthesis (P02746)	3	0.30%	1.50%
15	PDGF signaling pathway (P00047)	3	0.30%	1.50%
16	Muscarinic acetylcholine receptor 2 and 4 signaling pathway (P00043)	3	0.30%	1.50%
17	Muscarinic acetylcholine receptor 1 and 3 signaling pathway (P00042)	3	0.30%	1.50%
18	Metabotropic glutamate receptor group II pathway (P00040)	3	0.30%	1.50%
19	ATP synthesis (P02721)	3	0.30%	1.50%
20	Valine biosynthesis (P02785)	3	0.30%	1.50%
21	Huntington disease (P00029)	3	0.30%	1.50%
22	FGF signaling pathway (P00021)	3	0.30%	1.50%
23	CCKR signaling map (P06959)	2	0.20%	1.00%
24	Leucine biosynthesis (P02749)	2	0.20%	1.00%
25	Toll receptor signaling pathway (P00054)	2	0.20%	1.00%
26	De novo pyrimidine ribonucleotides biosythesis (P02740)	2	0.20%	1.00%
27	Arginine biosynthesis (P02728)	2	0.20%	1.00%
28	Tryptophan biosynthesis (P02783)	2	0.20%	1.00%

Supplementary table 1.

29	Endothelin signaling pathway (P00019)	2	0.20%	1.00%
30	Cell cycle (P00013)	2	0.20%	1.00%
31	B cell activation (P00010)	2	0.20%	1.00%
32	Pentose phosphate pathway (P02762)	2	0.20%	1.00%
33	SCW signaling pathway (P06216)	1	0.10%	0.50%
34	GBB signaling pathway (P06214)	1	0.10%	0.50%
35	DPP signaling pathway (P06213)	1	0.10%	0.50%
36	DPP-SCW signaling pathway (P06212)	1	0.10%	0.50%
37	BMP/activin signaling pathway-drosophila (P06211)	1	0.10%	0.50%
38	Gonadotropin-releasing hormone receptor pathway (P06664)	1	0.10%	0.50%
39	Pyridoxal-5-phosphate biosynthesis (P02759)	1	0.10%	0.50%
40	Angiogenesis (P00005)	1	0.10%	0.50%
41	Alzheimer disease-amyloid secretase pathway (P00003)	1	0.10%	0.50%
42	Mannose metabolism (P02752)	1	0.10%	0.50%
43	Lipoate_biosynthesis (P02750)	1	0.10%	0.50%
44	p53 pathway (P00059)	1	0.10%	0.50%
45	mRNA splicing (P00058)	1	0.10%	0.50%
46	Histidine biosynthesis (P02747)	1	0.10%	0.50%
47	VEGF signaling pathway (P00056)	1	0.10%	0.50%
48	Tetrahydrofolate biosynthesis (P02742)	1	0.10%	0.50%
49	TGF-beta signaling pathway (P00052)	1	0.10%	0.50%
50	Cysteine biosynthesis (P02737)	1	0.10%	0.50%
51	Notch signaling pathway (P00045)	1	0.10%	0.50%
52	Chorismate biosynthesis (P02734)	1	0.10%	0.50%
53	Synaptic vesicle trafficking (P05734)	1	0.10%	0.50%
54	Metabotropic glutamate receptor group III pathway (P00039)	1	0.10%	0.50%
55	Ionotropic glutamate receptor pathway (P00037)	1	0.10%	0.50%
56	Vitamin B6 metabolism (P02787)	1	0.10%	0.50%
57	Inflammation mediated by chemokine and cytokine signaling pathway (P00031)	1	0.10%	0.50%
58	Thyrotropin-releasing hormone receptor signaling pathway (P04394)	1	0.10%	0.50%
59	Oxytocin receptor mediated signaling pathway (P04391)	1	0.10%	0.50%
60	Heterotrimeric G-protein signaling pathway-Gi alpha and Gs alpha mediated pathway (P0	1	0.10%	0.50%
61	Hedgehog signaling pathway (P00025)	1	0.10%	0.50%
62	Sulfate assimilation (P02778)	1	0.10%	0.50%
63	General transcription by RNA polymerase I (P00022)	1	0.10%	0.50%
64	Enkephalin release (P05913)	1	0.10%	0.50%
65	Dopamine receptor mediated signaling pathway (P05912)	1	0.10%	0.50%
66	FAS signaling pathway (P00020)	1	0.10%	0.50%
67	Histamine H2 receptor mediated signaling pathway (P04386)	1	0.10%	0.50%
68	Pyruvate metabolism (P02772)	1	0.10%	0.50%

Supplementary table 1.

69	Histamine H1 receptor mediated signaling pathway (P04385)	1	0.10%	0.50%
70	Pyridoxal phosphate salvage pathway (P02770)	1	0.10%	0.50%
71	Purine metabolism (P02769)	1	0.10%	0.50%
72	Beta2 adrenergic receptor signaling pathway (P04378)	1	0.10%	0.50%
73	Beta1 adrenergic receptor signaling pathway (P04377)	1	0.10%	0.50%
74	5HT2 type receptor mediated signaling pathway (P04374)	1	0.10%	0.50%
75	5HT1 type receptor mediated signaling pathway (P04373)	1	0.10%	0.50%
Plasmodium falciparum				
1	Ubiquitin proteasome pathway (P00060)	8	1.50%	######
2	Huntington disease (P00029)	5	0.90%	6.30%
3	Transcription regulation by bZIP transcription factor (P00055)	4	0.80%	5.10%
4	General transcription regulation (P00023)	4	0.80%	5.10%
5	DNA replication (P00017)	4	0.80%	5.10%
6	Parkinson disease (P00049)	4	0.80%	5.10%
7	Wnt signaling pathway (P00057)	3	0.60%	3.80%
8	Nicotinic acetylcholine receptor signaling pathway (P00044)	3	0.60%	3.80%
9	p53 pathway (P00059)	2	0.40%	2.50%
10	mRNA splicing (P00058)	2	0.40%	2.50%
11	General transcription by RNA polymerase I (P00022)	2	0.40%	2.50%
12	Methylcitrate cycle (P02754)	2	0.40%	2.50%
13	Tryptophan biosynthesis (P02783)	2	0.40%	2.50%
14	CCKR signaling map (P06959)	2	0.40%	2.50%
15	De novo pyrimidine ribonucleotides biosythesis (P02740)	2	0.40%	2.50%
16	Pyruvate metabolism (P02772)	2	0.40%	2.50%
17	Gonadotropin-releasing hormone receptor pathway (P06664)	2	0.40%	2.50%
18	De novo purine biosynthesis (P02738)	1	0.20%	1.30%
19	Purine metabolism (P02769)	1	0.20%	1.30%
20	Alzheimer disease-presenilin pathway (P00004)	1	0.20%	1.30%
21	Interleukin signaling pathway (P00036)	1	0.20%	1.30%
22	Alzheimer disease-amyloid secretase pathway (P00003)	1	0.20%	1.30%
23	Adrenaline and noradrenaline biosynthesis (P00001)	1	0.20%	1.30%
24	Insulin/IGF pathway-protein kinase B signaling cascade (P00033)	1	0.20%	1.30%
25	Inflammation mediated by chemokine and cytokine signaling pathway (P00031)	1	0.20%	1.30%
26	p53 pathway feedback loops 2 (P04398)	1	0.20%	1.30%
27	Heterotrimeric G-protein signaling pathway-Gq alpha and Go alpha mediated pathway (P(	1	0.20%	1.30%
28	Heterotrimeric G-protein signaling pathway-Gi alpha and Gs alpha mediated pathway (P0(	1	0.20%	1.30%
29	Vitamin D metabolism and pathway (P04396)	1	0.20%	1.30%
30	Ras Pathway (P04393)	1	0.20%	1.30%
31	FGF signaling pathway (P00021)	1	0.20%	1.30%
32	FAS signaling pathway (P00020)	1	0.20%	1.30%

Supplementary table 1.

33	ATP synthesis (P02721)	1	0.20%	1.30%
34	TCA cycle (P00051)	1	0.20%	1.30%
35	Endothelin signaling pathway (P00019)	1	0.20%	1.30%
36	EGF receptor signaling pathway (P00018)	1	0.20%	1.30%
37	PI3 kinase pathway (P00048)	1	0.20%	1.30%
38	PDGF signaling pathway (P00047)	1	0.20%	1.30%
39	Heme biosynthesis (P02746)	1	0.20%	1.30%
40	Cell cycle (P00013)	1	0.20%	1.30%
41	Cadherin signaling pathway (P00012)	1	0.20%	1.30%
42	Muscarinic acetylcholine receptor 2 and 4 signaling pathway (P00043)	1	0.20%	1.30%
43	Salvage pyrimidine ribonucleotides (P02775)	1	0.20%	1.30%
