# Supplementary material for: Large-Scale Analyses of Site-Specific Evolutionary Rates across Eukaryote Proteomes Reveal Confounding Interactions between Intrinsic Disorder, Secondary Structure, and Functional Domains
Source: Genes (Basel). 2018 Nov 14;9(11):553. doi: 10.3390/genes9110553 (PMC6265720; doi:10.3390/genes9110553)
Supplement: Supplementary file 1 [file genes-09-00553-s001.zip › Supplementary_table_2.pdf]

Supplementary Table 2 – Nonparametric *Post Hoc* Multiple Comparison Results.

Supplementary Table 2

Nonparametric *Post Hoc* Multiple Comparison Results				
(TURE = significant; Corrected Alpha = 0.05)				
Comparison	Metazoans	Plants	Saccharomycetes	Alveolates
Disordered-Coil-Domain vs. Disordered-Coil-Linker	TRUE	TRUE	TRUE	TRUE
Disordered-Coil-Domain vs. Disordered-Structure-Domai	FALSE	FALSE	FALSE	TRUE
Disordered-Coil-Domain vs. Disordered-Structure-Linker	TRUE	TRUE	FALSE	TRUE
Disordered-Coil-Domain vs. Ordered-Coil-Domain	TRUE	TRUE	TRUE	FALSE
Disordered-Coil-Domain vs. Ordered-Coil-Linker	TRUE	TRUE	TRUE	FALSE
Disordered-Coil-Domain vs. Ordered-Structure-Domain	TRUE	TRUE	TRUE	TRUE
Disordered-Coil-Domain vs. Ordered-Structure-Linker	TRUE	TRUE	TRUE	TRUE
Disordered-Coil-Linker vs. Disordered-Structure-Domain	TRUE	TRUE	TRUE	TRUE
Disordered-Coil-Linker vs. Disordered-Structure-Linker	TRUE	FALSE	TRUE	TRUE
Disordered-Coil-Linker vs. Ordered-Coil-Domain	TRUE	TRUE	TRUE	TRUE
Disordered-Coil-Linker vs. Ordered-Coil-Linker	TRUE	TRUE	TRUE	TRUE
Disordered-Coil-Linker vs. Ordered-Structure-Domain	TRUE	TRUE	TRUE	TRUE
Disordered-Coil-Linker vs. Ordered-Structure-Linker	TRUE	TRUE	TRUE	TRUE
sordered-Structure-Domain vs. Disordered-Structure-Link	TRUE	TRUE	FALSE	TRUE
Disordered-Structure-Domain vs. Ordered-Coil-Domain	FALSE	TRUE	FALSE	TRUE
Disordered-Structure-Domain vs. Ordered-Coil-Linker	TRUE	TRUE	FALSE	TRUE
isordered-Structure-Domain vs. Ordered-Structure-Doma	TRUE	TRUE	TRUE	FALSE
Disordered-Structure-Domain vs. Ordered-Structure-Linke	TRUE	TRUE	TRUE	TRUE
Disordered-Structure-Linker vs. Ordered-Coil-Domain	TRUE	TRUE	FALSE	TRUE
Disordered-Structure-Linker vs. Ordered-Coil-Linker	FALSE	TRUE	FALSE	FALSE
Disordered-Structure-Linker vs. Ordered-Structure-Domai	FALSE	TRUE	TRUE	TRUE
Disordered-Structure-Linker vs. Ordered-Structure-Linker	TRUE	TRUE	TRUE	FALSE
Ordered-Coil-Domain vs. Ordered-Coil-Linker	TRUE	TRUE	FALSE	TRUE
Ordered-Coil-Domain vs. Ordered-Structure-Domain	TRUE	TRUE	TRUE	TRUE
Ordered-Coil-Domain vs. Ordered-Structure-Linker	TRUE	TRUE	TRUE	TRUE
Ordered-Coil-Linker vs. Ordered-Structure-Domain	TRUE	TRUE	TRUE	TRUE
Ordered-Coil-Linker vs. Ordered-Structure-Linker	TRUE	TRUE	TRUE	TRUE
Ordered-Structure-Domain vs. Ordered-Structure-Linker	TRUE	TRUE	FALSE	TRUE
